# Altered Mental Status and Cyanosis in a Pediatric Patient with Methemoglobinemia

**DOI:** 10.1155/2020/8896754

**Published:** 2020-11-20

**Authors:** Phillip M. Grenz, Robert N. Ray Jr., Olivia A. Hardy, Andrew L. Koons, Kenneth D. Katz, Teresa M. Romano

**Affiliations:** ^1^Lehigh Valley Health Network, Department of Emergency and Hospital Medicine, USF Morsani College of Medicine, Cedar Crest Boulevard & I-78, Allentown 18103, PA, USA; ^2^Lehigh Valley Health Network, Department of Emergency and Hospital Medicine, Division of Toxicology, USF Morsani College of Medicine, Cedar Crest Boulevard & I-78, Allentown 18103, PA, USA; ^3^Lehigh Valley Health Network, Department of Emergency and Hospital Medicine, Division of Pediatric Emergency Medicine, USF Morsani College of Medicine, Cedar Crest Boulevard & I-78, Allentown 18103, PA, USA

## Abstract

Methemoglobinemia results from increased amounts of oxidized hemoglobin in the blood with an ensuing change in oxygen dissociation curve and lack of oxygen delivery to tissue. A previously well, male toddler was brought to the Pediatric Emergency Department (PED) by Emergency Medical Services (EMS) with abrupt onset of altered mental status and cyanosis after a suspected ingestion of “Rush” nail polish remover. He was quickly diagnosed with methemoglobinemia by both clinical presentation and chocolate-colored blood appearance. He emergently received intravenous (IV) methylene blue (MB) with immediate and sustained improvement requiring no further doses. Though inhalation of nitrites and subsequent methemoglobinemia is frequently reported in adolescents, we were unable to find any cases in the literature detailing ingestion of this product and the resulting clinical manifestations. Our objective with this report is to describe a rare case of a toddler with an accidental ingestion of “Rush” nail polish remover, a nitrite compound. Our patient presented to the PED with abrupt onset of altered level of consciousness, hypotension, and cyanosis resulting from acquired methemoglobinemia. This case report demonstrates the importance of emergency clinicians being able to make clinical judgements and decisions based on the history and physical exam when methemoglobinemia is suspected.

## 1. Introduction

Methemoglobinemia results from increased amounts of oxidized hemoglobin in the blood with an ensuing change in oxygen dissociation curve and lack of oxygen delivery to tissue [1, 2]. Ultimately, this can cause cyanosis, dyspnea, headache, fatigue, weakness, seizures, metabolic acidosis, coma, and death [3]. The severity of the signs and symptoms often correlates with the percent of methemoglobin in the blood, with 1-2% naturally occurring [1, 3]. Common oxidizing agents include nitrite compounds, dapsone, chloroquine and quinones, aniline dyes, and some local anesthetics [4]. Acquired methemoglobinemia is not a common cause of cyanosis in the Pediatric Emergency Department (PED) [5]. Our objective of this report is to describe a rare case of a toddler with an accidental ingestion of “Rush” nail polish remover, a nitrite compound. He presented to the PED with abrupt onset of altered level of consciousness, hypotension, and cyanosis resulting from acquired methemoglobinemia.

## 2. Case Presentation

**Figure 1:**
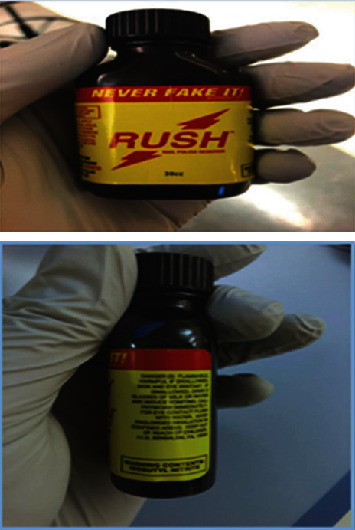
“Rush” nail polish remover.

A previously well, 2-year-old male toddler was brought to the Pediatric Emergency Department (PED) by Emergency Medical Services (EMS) for altered mental status and hypotension after a suspected ingestion while playing unsupervised at home. The mother noticed that he became abnormally quiet, and when she checked on him, he complained of a “hot” sensation in his throat. The mother discovered a family member's empty bottle of “Rush” nail polish remover (Figure 1) with a loose lid and smelled an aroma of nail polish remover on his breath. EMS was called and on arrival to the patient's home, noted hypoxia and altered mental status. The patient received nasal cannula oxygen en route to the hospital and remained unusually subdued throughout transport. He arrived in the PED with the bottle approximately 45 minutes after the exposure, with profound pallor and persistent hypoxia without associated respiratory distress. The only chemical ingredient listed on the bottle was isobutyl nitrite. The family member to whom the bottle belonged stated that it was used as a sexual enhancement supplement.

**Figure 2:**
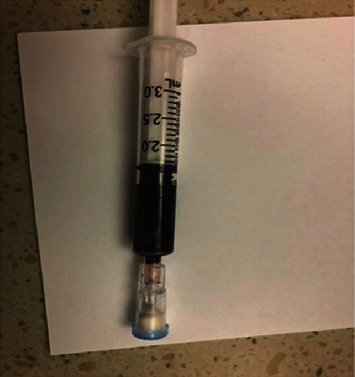
Patient's initial blood sample.

In the PED, the child appeared critically ill, exhibiting lethargy, hypotension, and pallor. His extremities, albeit pale, were not cool to the touch, and he had perioral cyanosis. His initial vital signs were pulseoximetry, 90% (1 liter nasal cannula); heart rate, 160 beats per minute; respiratory rate, 40 respirations per minute; and blood pressure, 69/56 mmHg. He continued to demonstrate hypoxia with a pulseoximetry of 86–90% despite 2 liters of oxygen nasal cannula. Despite this hypoxia, he did not demonstrate significant tachypnea or increased work of breathing, and his breath sounds on lung auscultation remained clear. Immediate IV access was obtained, and his blood sample was thick and chocolate-brown in color (Figure 2). Based on the clinical presentation and exposure, the presumptive diagnosis of methemoglobinemia was made, and the patient's blood sample was sent for co-oximetry and additional laboratory studies. 2 mg/kg (24 mg) IV methylene blue was administered. Within minutes, the patient's vital signs, lethargy, and cyanosis resolved. The initial venous blood gas (VBG) measured pH, 7.12; pCO_2_, 54 mmHg; pO_2_, < 48 mmHg; and HCO_3_, 18 mEq/L (normal values: pH, 7.35–7.45; pCO_2_, 41–51 mmHg; pO_2_, normal range not established for VBGs; and HCO_3_, 23–29 mEq/L). The initial serum lactate measured 6.9 mmol/L. A comprehensive metabolic panel (CMP) revealed HCO_3_ of 19 mmol/L and creatinine of 1.18 mg/dL but was otherwise normal. The anion gap was 12, and the complete blood count, liver function tests, urinalysis, coagulation studies, electrocardiogram, and chest X-ray were all normal. Acetaminophen, salicylate, ethanol, ethylene glycol, isopropanol, methanol, and urine drug screen were all negative. Three attempts were made to send initial blood samples for co-oximetry, but persistent laboratory machine error likely due to the extreme color of the blood did not allow the machines to obtain any reading or methemoglobin level. The samples were sent to co-oximetry in the PED as well as two samples to the hospital's main laboratory. Methylene blue was administered, and labs were repeated which showed a marked improvement within hours. The lactate normalized to 1.5 mmol/L, and the CMP showed CO_2_ of 21 mmol/L and creatinine of 0.53 mg/dL.

The patient was then admitted to the hospital. On hospital day 2, co-oximetry testing measured a methemoglobin of 1%. The patient was discharged home later that same day in normal condition. The family ensured that all potential toxins would be unequivocally removed from the child's environment.

## 3. Discussion

Methemoglobinemia refers to the ferric form of hemoglobin (Fe^3+^), and this occurs when a chemical or drug oxidizes the ferrous hemoglobin (Fe^2+^). Fe^3+^ hemoglobin is incapable of carrying oxygen causing a functional anemia and shifting the oxygen dissociation curve to the left. The signs and symptoms of methemoglobinemia occur from the inability of hemoglobin to bind and subsequently deliver oxygen to the tissues [2].

There are a variety of medications that can induce methemoglobinemia, and the most commonly reported is the local anesthetic benzocaine—often used in endoscopic procedures. It can also be induced by nitrates and nitrites, antibiotics such as dapsone or trimethoprim-sulfamethoxazole, and other drugs such as metoclopramide. In addition, methemoglobinemia can be induced by exposure to chemicals such as fertilizers, weed killers, dyes, and some paints [6].

The clinical presentation of methemoglobinemia correlates with the percent of hemoglobin that has been oxidized. At <15% methemoglobin, healthy patients are generally asymptomatic, and the course is self-limited. At 20–30%, patients become symptomatic including mental status changes, headache, fatigue, exercise intolerance, dizziness, and syncope. At levels >50%, patients can have severe symptoms including acidosis, elevated lactate values, dysrhythmias, seizures, coma, and death [7].

Methemoglobinemia can be diagnosed clinically. Typical scenarios include cyanotic patients with refractory hypoxemia after starting a new medication, a suspected or known ingestion, or after a procedure which required local anesthetic. The pulseoximetry will commonly read in the mid to high 80s despite maximal oxygen therapy. In addition, the patient's blood will appear “chocolate-brown”. The diagnosis can be confirmed with certain laboratory tests including co-oximetry which will provide a percentage of methemoglobin in the blood. Other helpful data may include a blood gas that demonstrates metabolic acidosis and a lactate which may be elevated.

Treatment of methemoglobinemia is with the antidote methylene blue. The dose is 1-2 mg/kg intravenously over five minutes and is contraindicated in glucose-6-phosphate dehydrogenase (G6PD) deficiency. It is indicated in patients who have moderate or severe manifestations or if the methemoglobin levels are greater than 25–30%. Repeat doses can be given hourly for recurrent symptoms or refractory methemoglobin percentages. It is important to note that methylene blue will appear to cause the oxygen saturation to drop significantly. This is not true hypoxia but the nature of the pulse oximeter's interpretation of the color changes that the antidote causes in the blood. It is also interesting to note that body secretions such as tears, urine, and vomit may turn blue for several hours after methylene blue administration. Methylene blue works by acting as an electron shuttle utilizing NADPH and G6PD to reduce methemoglobin to normal hemoglobin [8].

### 3.1. Diagnostic Limitations

Methemoglobinemia is an uncommon cause of cyanosis in the PED, and the diagnosis may present a challenge. The pediatric population can also present with cyanosis from maladies such as congenital heart disease, pulmonary and airway abnormalities, other hemoglobinopathies, and sepsis. Moreover, pulseoximetry detects only oxyhemoglobin and deoxyhemoglobin rather than methemoglobin and generally do not change with supplemental oxygen administration similar to shunt physiology; pulseoximetry readings can measure as high as 85% in the presence of 30% MetHgb [9]. Arterial blood gas is similarly flawed to diagnose MetHgb as it measures the partial pressure of dissolved oxygen (PaO_2_) rather than oxygen bound to hemoglobin [2]. Therefore, PaO_2_ may be normal in MetHgb even though there is very little hemoglobin-bound O_2_ available for oxygen delivery to tissues. The gold standard for diagnosing methemoglobinemia is co-oximetry, which measures the concentration of different types of hemoglobin in the blood by spectrophotometry and wavelength measurement [10]. It is possible, as was seen in this case, that co-oximetry will not be able to determine methemoglobin concentration depending on the characteristics of the blood sample. This further illustrates the importance of being able to recognize this diagnosis clinically.

This case was limited by the lack of a methemoglobin value to go with the clinical picture of methemoglobinemia. The child's blood sample was run on multiple machines both in the PED and in the hospital laboratory. Results were reported out as “unreadable,” or included an error message in all attempts during the acute management. The child's presentation and response to methylene blue clearly supports the diagnosis. However, this case highlights that, in some extreme instances, the co-oximetry testing may be faulty due to the constitution of the blood sample.

### 3.2. Conclusions

This case report demonstrates the importance of emergency clinicians being able to make clinical judgements and decisions based on the history and physical exam when methemoglobinemia is suspected.

## Data Availability

Queries regarding availability of references and source image data used to support the findings of this study can be directed to the corresponding author.
